# Diagnostic performance of magnetic resonance imaging and 3D endoanal ultrasound in detection, staging and assessment post treatment, in anal cancer

**DOI:** 10.18632/oncotarget.14946

**Published:** 2017-02-01

**Authors:** Alfonso Reginelli, Vincenza Granata, Roberta Fusco, Francesco Granata, Daniela Rega, Luca Roberto, Gianluca Pellino, Antonio Rotondo, Francesco Selvaggi, Francesco Izzo, Antonella Petrillo, Roberto Grassi

**Affiliations:** ^1^ Department of Internal and Experimental Medicine, Magrassi-Lanzara, Institute of Radiology, Second University of Naples, Naples, Italy; ^2^ Department of Diagnostic Imaging, Radiant and Metabolic Therapy, “Istituto Nazionale Tumori Fondazione Giovanni Pascale – IRCCS”, Naples, Italy; ^3^ Departement of Civil and Mechanical Engineering, University of Cassino and Southern Lazio, Cassino, Italy; ^4^ Department of Colorectal Surgical Oncology, “Istituto Nazionale Tumori Fondazione Giovanni Pascale – IRCCS”, Naples, Italy; ^5^ Department of Medical, Surgical, Neurological, Metabolic and Ageing Sciences, Second University of Naples, Naples, Italy; ^6^ Department of Surgical Oncology, “Istituto Nazionale Tumori Fondazione Giovanni Pascale – IRCCS”, Naples, Italy

**Keywords:** anal cancer, 3D endo anal ultrasound, magnetic resonance imaging, diagnostic performance, post-treatment imaging assessment

## Abstract

We compared Magnetic Resonance Imaging (MRI) and 3D Endoanal Ultrasound (EAUS) imaging performance to confirm anal carcinoma and to monitor treatment response.

58 patients with anal cancer were retrospectively enrolled. All patients underwent clinical examination, anoscopic examination; EAUS and contrast-enhanced MRI study before and after treatment. Four radiologists evaluated the presence of lesions, using a 4-point confidence scale, features of the lesion and nodes on EAUS images, T1-weighted (T1-W), T2-weighted (T2-W) and diffusion-weighted images (DWI) signal intensity (SI), the apparent diffusion coefficient (ADC) map for nodes and lesion, as well as enhancement pattern during dynamic MRI were assessed.

All lesions were detected by EAUS while MRI detected 93.1% of anal cancer. MRI showed a good correlation with EAUS, anoscopy and clinical examination. The residual tissue not showed significant difference in EAUS assessment and T2-W SI in pre and post treatment. We found significant difference in dynamic study, in SI of DWI, in ADC map and values among responder's patients in pre and post treatment. The neoplastic nodes were hypoecoic on EAUS, with hyperintense signal on T2-W sequences and hypointense signal on T1-W. The neoplastic nodes showed SI on DWI sequences and ADC value similar to anal cancer. We found significant difference in nodes status in pre and post therapy on DWI data.

3D EAUS and MRI are accurate techniques in anal cancer staging, although EAUS is more accurate than MRI for T1 stage. MRI allows correct detection of neoplastic nodes and can properly stratify patients into responders or non responders.

## INTRODUCTION

Anal carcinoma is a rare malignancy with an incidence of 2 new cases per 100,000 per year in the USA [1], accounting approximately 0.4% of all tumors and 2.5% of gastrointestinal malignancies [2–4]. Risk factors associated are the number of sexual partners, genital warts, vulvar, vaginal or cervical cancer, and viral infections by human papillomavirus (HPV), and human immunodeficiency virus (HIV) [3–6]. The diagnosis based only on history and clinical data is difficult since the symptoms reported by patients are similar to those with benign diseases: the 45 % of patients report rectal bleeding, 20–35% anorectal pain and 20-35% sensation of a rectal mass [7–8]. Proper recognition of the anal cancer is crucial for the patient management, whereas an early detection allows conservative treatment with a reserve of sphincter function [9]. The recent improvements of radiotherapy and chemotherapy as neoadjuvant therapies, can also down staging the lesion, as well as to allow a conservative treatment [9–11]. According to National Comprehensive Cancer Network (NCCN) Anal Carcinoma Guidelines the patients should be subjected to a careful clinical examination, including a digital rectal examination (DRE), an anoscopic examination, and palpation of inguinal nodes, to evaluate T stage, while the role of Computed Tomography (CT) and Magnetic Resonance Imaging (MRI) is limited to the identification of regional nodes, the endoanalultrasound (EAUS) is not recommended [12]. The EAUS and MRI allow a detailed evaluation of the multilayer wall of the ano, sphincter plan, relations with adjacent structures and the presence of lymphadenopathy, which is mandatory in staging, to identify a correct therapeutic strategy. Three Dimensional (3D) -EAUS is a valuable tool to represent the normal anatomy and diseases of the anal canal. It is easy to perform and to reproduce, painless, with high diagnostic accuracy. It provides excellent imaging of the anal wall, of the internal and external sphincters and of the intersphincteric plane, essential for planning surgical approach [13]. 3D-EAUS is the technique of choice in benign anal diseases [14]. MRI is the gold standard in oncological pelvic examination, providing morphological and functional data [15]. Moreover, MR imaging plays an important role in therapeutic assessment, properly stratify patients into responders or non-responders to neoadjuvant treatment, in surveillance after surgery, and in recurrence [15–16].

Objective of our study is to compare the diagnostic performance of EUS and MRI in the detection, staging and assessment of anal cancer patients after therapy.

## RESULTS

Clinical examination and anoscopic examination identified 58 patients (35 women and 23 men, mean age 53, range 42-73) with squamous cell carcinomas. All lesions were histological proven. 37 (63.8%) lesions involving distal anal channel and 21 (36.2%) involving proximal anal channel. According to clinical staging (TNM) [20]: 4 (6.9%) lesions were T1, 2 (3.4%) were T2, 39 (67.2%) were T3, and 13 (22.4%) were T4 [3].

All lesions were detected by EAUS, and the correlation in the T stage identification between EAUS and clinical and anoscopic examination was 100%: 4 (6.9%) lesions were T1, 2 (3.4%) were T2, 39 (67.2%) were T3, and 13 (22.4%) were T4.

MRI detected 54/58 (93.1%) anal cancer; the undetected lesions were all T1 stage. Also MRI showed a good correlation with EAUS, anoscopy and clinical examination when the stage was greater than T1: 2 (3.4%) were T2, 39 (67.2%) were T3, and 13 (22.4%) were T4. According to the confidence scale, for lesion detection, the median value obtained was 4 for EAUS; 3.8 for TSE T2-W sequences, 3.6 for DWI and ADC maps; 3.8 for Flash T1-W GRE dynamic study and 3.8 for T1-W TSE post contrast medium.

**Figure 1:**
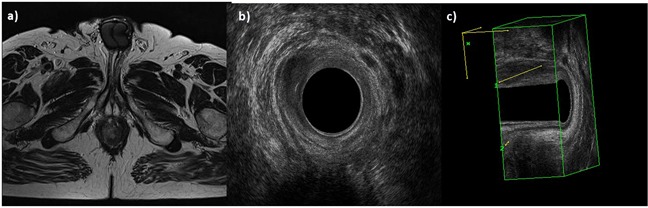
Man 42y, anal cancer In **a**. TSE T2-W in axial plane, the lesion infiltrates internal and external sphincters, as showed also by 2 D **b**. and 3D **c**. EAUS.

EAUS and MRI identified the involvement of anal verge in 28 (48.3%) patients, of anorectal junction in 17 (29.3%) patients, of internal sphincter in 54 (93.0%) patients (Figure 1) and of external sphincter in 47 (81.0%), with a correlation of 100%.

EAUS detected the presence of lymphadenopathy in 39 (67.2%) patients in mesorectal fat. MRI identified the presence of lymphadenopathy in 46 (79.3%) patients in mesorectal fat and in 44 (76.0%) patients in inguinal and iliac site. In Table 1 we report the patients stage according to EAUS, MRI and clinical and anoscopic data.

**Table 1:** Anatomic patients stage

STAGE	Numbers (%)
Stage 0	0 (0.0%)
Stage I	4 (6.9%)
Stage II	2 (3.45%)
Stage IIIA	7 (12.1%)
Stage IIIB	44 (75.8%)
Stage IV	1 (1.7%)

All lesions were hypoecoic on EAUS.

**Figure 2:**
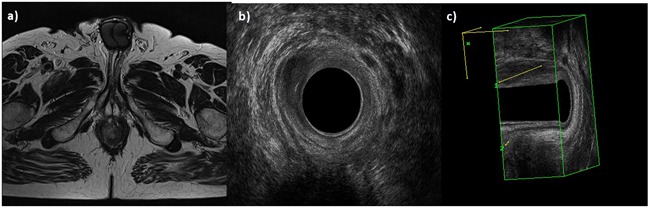
Man 54y, anal cancer In **a**. TSE T2-W in axial plane, the lesion infiltrates only internal sphincter, as showed also by 2 D **b**. and 3D **c**. EAUS.

The lesions showed hyperintense signal on T2-W (Figure 2) and hypointense signal on T1-W. The diffusion was restricted from *b*0 s/mm^2^ to *b*800 s/mm^2^, with hyperintense signal on b800 s/mm^2^, hypointense signal on ADC map and the median ADC value was of 830×10^−3^ mm^2^/s (range, 760-904×10^−3^ mm^2^/s). All lesions showed a TIC type 3, with rapid initial and sustained late enhancement. In Table 2 we have summarized the aspects of the lesions on MR study.

**Table 2:** MRI anal cancer features

Sequences	SI/ median value/ type
T2-W	Hyperintense
T1-W	Hypointense
DWI	Hyperintense
ADC map	Hypointense
ADC	830 x10^−3^ mm^2^/s
TIC	Type 3

On MR T2-W sequence, the median measure of perianal nodes was 5 mm (range 3-7 mm); of perirectal nodes was 8 mm (range 6-14 mm); of iliac nodes was 17 mm (range 12-21mm) and of inguinal nodes was 24 mm (range 16-34 mm). On EAUS the median measure of perianal nodes was 5 mm (range 3-7 mm). We no found differences between MRI and EAUS assessment of the perianal nodes (p = 0.35 at Mann–Whitney U test).

The neoplastic nodes were hypoecoic on EAUS, with hyperintense signal on T2-W sequences and hypointense signal on T1-W, with restricted diffusion (from *b*0 s/mm^2^ to *b*800 s/mm^2^). The signal was hyperintense on b800 s/mm^2^, hypointense on ADC map and the median ADC value was of 790×10^−3^ mm^2^/s (range, 738-892×10^−3^ mm^2^/s). The neoplastic nodes showed SI on DWI sequences and the ADC value was similar to anal cancer, with an overlapping of ADC values.

Four patients underwent surgery; 54 patients underwent neoadjuvant therapy: 44 patients were responders to therapy and 10 were non-responder to therapy.

Both techniques showed a significant reduction in the lesion size in the post-treatment examinations, as well as a disappearance or a reduction in the size of lymphadenopathy.

**Figure 3:**
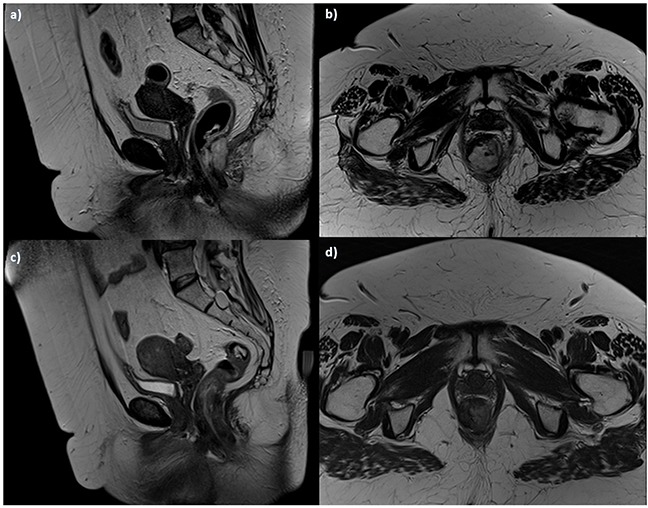
Woman 54y, anal cancer TSE T2-W in sagittal **a**. and axial **b**. plane, pre-treatment morphological assessment: the lesion infiltrates internal and external sphincters. Post treatment assessment: TSE T2-W images in sagittal **c**. and axial **d**. plane show tumor size reduction, with residual tissue.

**Figure 4:**
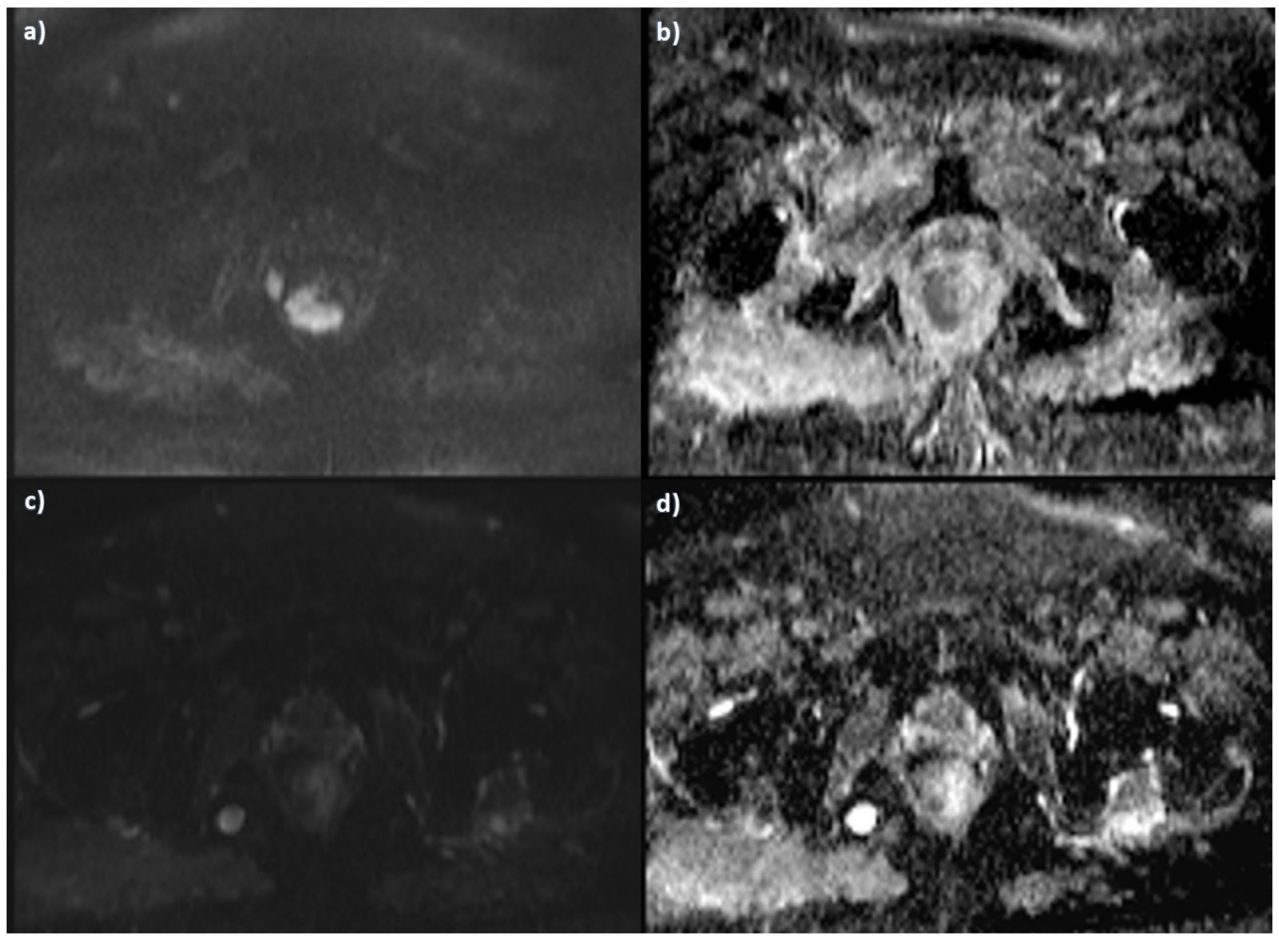
The same patient of Figure 3 Pre-treatment DWI images, in **a**. b800 s/mm2 the lesion shows restricted signal in **b**. ADC map with hypointense signal of the lesion. Post treatment assessment with a lower SI in b 800 s/mm2 **c**. and higher SI in ADC map **d**.

**Figure 5:**
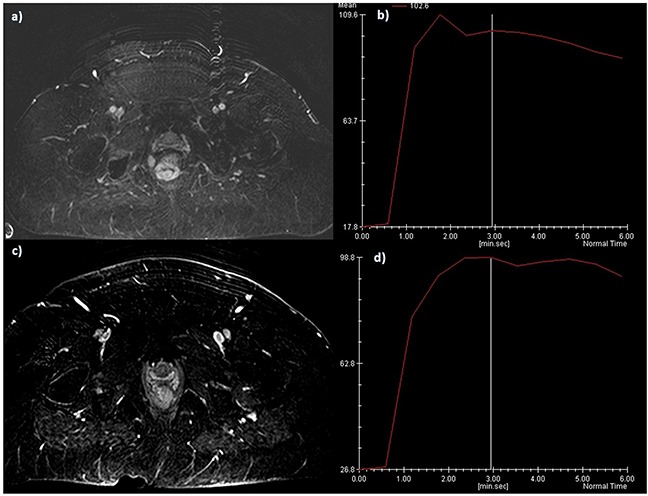
The same patient of Figure 3 and D, DCE-MRI and TIC Pre treatment evaluation: in **a**. subtraction image and in **b**. type 3 curve. Post treatment evaluation in **c**. subtraction image and in **d**. type 2 curve. Responder patient.

When we analyzed the residual anal tissue after treatment, we found no significant difference in EUS assessment and T2-W SI between pre and post treatment both for responders (p = 0.11 at Wilcoxon test) and for non responders patients (p = 0.32 at Wilcoxon test) (Figure 3): residual cancer and fibrosis showed similar hypoechoic appearance and hyperintense signal. Conversely we found significant difference in dynamic study with correlate inspective analysis of TIC (type 2, slow sustained enhancement), in SI of DWI (less restriction of water diffusion with less hyperintensity in b800 s/mm^2^) and of ADC map (less hypointensity) and ADC values (1220×10^−3^ mm^2^/s; range 910-1310×10^−3^ mm^2^/s) between pre and post treatment for responders patients (p = 0.02, 0.03, 0.001,0.003 respectively at Wilcoxon test) (Figure 4 and Figure 5). While for non-responders patients TIC, SI of DWI and ADC map and ADC values not showed significant differences between pre and post treatment (p = 0.23 at Wilcoxon test).

In responders patients the median measure of nodes has shrunk more than 30%, with many lymph nodes that were not detect in post treatment examination. On MR T2-W sequence and EAUS, the median measure of perianal nodes was 1.5 mm (range 0.2-2 mm); of perirectal nodes was 2.1 mm (range 3-8 mm); of iliac nodes was 5 mm (range 3-8 mm) and of inguinal nodes was 7 mm (range 5-12 mm). We found significant difference in nodes status between pre and post therapy on DWI data: the SI showed a less restriction of water diffusion with less hyperintensity or disappearance in b800 s/mm2; a SI on ADC map less hypointensity and an increase of ADC values (1180×10^−3^ mm^2^/s; range 1101-1203×10^−3^ mm^2^/s) with a p = 0.02 at Wilcoxon test.

In Table 3 we summarized our results.

**Table 3:** Summary of results

Technique	3D EAUS	MRI
Tumor detection	100% patients; Any T	54/58 (93.1%) patients;the undetected lesions were all T1 stage
Involvement of anal verge	28 (48.3%) patients	28 (48.3%) patients
Involvement of internal sphincter	54 (93%) patients	54 (93%) patients
Involvement of external sphincter	47 (81%) patients	47 (81%) patients
Detection of nodes in perianal and in mesorectal fat	39 (67.2%) patients;the median measure of perianal nodes was 5 mm (range 3-7 mm) and of perirectal nodes was 8 mm (range 6-14 mm)	46 (79.3%) patients; the median measure of perianal nodes was 5 mm (range 3-7 mm) and of perirectal nodes was 8 mm (range 6-14 mm)
Detection of nodes in inguinal and iliac site	0 (0%) patients	44 (76.0%) patients; The median measure of iliac nodes was 17 mm (range 12-21mm) and of inguinal nodes was 24 mm (range 16-34 mm).
Cancer assessment post therapy	No difference between residual cancer and fibrosis	Significant difference in DCE-MRI with analysis of TIC (type 2), in DWI (less restriction with less hyperintensity in b800 s/mm2) and of ADC map (less hypointensity) and ADC values (1220×10-3 mm2/s; range 910-1310×10-3 mm2/s) for responders patients between pre and post treatment. For non responders patients TIC, SI of DWI and ADC map and ADC values not showed significant differences between pre and post treatment
Nodes assessment post therapy	For responder patients the median measure of perianal nodes was 1,5 mm (range 0.2-2 mm)	In responder patients the median measure of perianal nodes was 1.5 mm (range 0,2-2 mm); of perirectal nodes was 2,1 mm (range 3-8 mm); of iliac nodes was 5 mm (range 3-8 mm) and of inguinal nodes was 7 mm (range 5-12 mm). Significant difference in nodes status between pre and post therapy on DWI data: the SI less hyperintensity or disappearance in b800 s/mm2; a SI on ADC map less hypointensity and an increase of ADC values (1180×10-3 mm2/s; range 1101-1203×10-3 mm2/s)

## DISCUSSION AND CONCLUSION

To the best of our knowledge, this is the first study which analyzes a so large group of patients with anal cancer, assessing not only the diagnostic performance of 3D-EAUS and MR in the detection and staging of the lesion, but also for the response to neoadjuvant treatment. Although, as recommended by the NCCN [12], the clinic, anoscopic and histologic examination, correctly identifies the presence of the lesion, with a 100% agreement with EAUS, as we showed, for all T stage, and with MRI for stage higher than T1, however, they are not adequate to identify prognostic factors, such as the iliac lymphadenopathy or the involvement of structures such as the sphincter level, rather than the vagina, that deeply modify the patient's management [3-5; 17-22]. Few studies evaluated the role of 3D EAUS in anal cancer [23–27]. According to Kolev et al [23], that demonstrated that T category on 3-D EAUS correlated with histopathology in 92.9%, and N category correlated with histopathology in 81.6%, our results showed that 3D EAUS is a valuable diagnostic tool in the assessment of T stage, even for stage T1. Christensen et al in 2004 compared 3-D endosonography with 2-D endosonography showed that 3D EAUS improved detection of perirectal lymph nodes becoming a powerful tool in staging and planning of treatment [24]. Our results, conversely to [24], showed as EAUS detected the presence of lymphadenopathy only in 39 (67.2%) patients in mesorectal fat while MRI identified the presence of lymphadenopathy in 46 (79.3%) patients, due to the largest field of view of MR study. Christensen et al in 2006 [25] showed that 3D EAUS was an accurate technique in detection of recurrence of anal cancer in combination with anoscopy and digital rectal examination; in our study we did not enrolled patients with recurrence disease and this is an our limit. Fewer studies are known on the evaluation of treatment and follow-up post surgery [25, 26]. Peterson et al, conversely to [25] demonstrated that EAUS did not provide any advantage over DRE in identifying locally recurrent disease, and should not be recommended for routine surveillance [26]. We evaluated the rule of 3D EAUS post neoadjuvant therapy showing that on 3D EAUS images, a significant reduction in the lesion size in post-treatment examinations, as well as a disappearance of lymphadenopathy was found. However, the technique was not able to differentiate the residual tissue as cancer or fibrosis. MRI is the gold standard in oncological rectal examination, providing morphological and functional data. MR provides preoperative assessment of important prognostic outlines, which may guide patient selection for neoadjuvant therapies; moreover, MR imaging plays an important role in therapeutic assessment [15–16]. Our results demonstrated how also in the anal cancer the MRI is a valuable diagnostic tool, although the major limitation is an incorrect detection of T1 patients, neither would seem that the use of the endoanal coil could increase the detection rate [27]. In fact Matsuoka et al showed that endorectal coil and phased array coil showed similar diagnostic accuracy in detection of anal cancer [27]. Several studies evaluated the MRI accuracy compared to EAUS, in rectal cancer patients staging, and the data suggested that EAUS provides an excellent visualization of the layers of the bowel wall conversely to MR so EAUS provides better detection of superficial tumor [23, 27]. In the evaluation of perianal and perirectal nodes, the techniques are complementary tool, while MR is the primary choice to identify iliac and inguinal nodes. According to Burdan et al [13], the possibility to obtain functional data by MR as the increased signal on DWI and low ADC values seem to predict the involvement of pelvic nodes better than their size alone. In fact we identified not enlarged nodes on DW images confirmed as neoplastic to histological examination.

Although the only morphological data both 3D EAUS and MRI, had allowed identify a patient as a responder to treatment, these did not allow to characterize the residual tissue, conversely by functional analysis. In fact, the most interesting aspect of our study is the functional evaluation of residual tissue post treatment. We analyzed the data of DWI and DCE-MRI found significant difference on residual tissue in responders: in Dynamic study the type 3 TIC became type 2 TIC and SI of lesion on DWI in b800 s/mm^2^ became less hyperintense with higher ADC (1220×10^−3^ mm^2^/s) compared to ADC pre treatment (830×10^−3^ mm^2^/s). These data suggested that residual tissue was fibrotic or inflammatory; while in non-responder patients the functional DWI and DCE-MR data were similar before and after treatment with an overlapping. Also for nodes status in pre and post therapy the DWI data showed a less restriction of water diffusion with less hyperintensity or disappearance in b800 s/mm2, a SI on ADC map less hypointensity with an increase of ADC values in responders patients. To the best of our knowledge, there are not studies that evaluated these features. However we think there will be need of more functional study to make this data robust. Also Goh et al evaluated the MRI pre and post treatment showed that early assessment of response by MRI at 6-8 weeks is unhelpful in predicting future clinical outcome [28–29]. In this study they considered only RECIST criteria, differently from them we evaluated functional data, but our limit is that we did not correlated the data with clinical outcome.

Although the anal caner is a rare neoplasm, the incidence of the tumor shows an incremental trend, and the real rule of imaging techniques in detection, staging and follow-up of this tumor should be cleared. 3D EAUS and MRI are a valuable diagnostic tools in detection of anal cancer, although we demonstrated that 3D EAUS is more accurate than MRI for T1 stage. On the other hand the MRI allows a correct detection of neoplastic nodes both to higher field of view and by functional data. In our study the possibility to obtain functional data by MR due to increased signal on DWI and low ADC values, allowed us to predict the involvement of pelvic nodes even when the size were not enlarged. Our results also suggested that MRI is the technique of choice post neoadjuvant treatment, because allow to properly stratify patients into responders or non-responders thanks to the assessment of functional data obtained by DCE-MRI (TIC) and DWI (SI and ADC values); while TIC and ADC values allow to characterize the residual tissue as cancer or fibrosis as well as ADC can identify if a nodes responded to therapy.

## MATERIALS AND METHODS

The study was approved by our ethics committee. Informed consent for both 3D EAUS and MRI was obtained in writing from all patients. All the data were collected and managed according to the privacy regulation in our country. Radiologists performed all 3D EAUS examinations.

### Patient population

From May 2010 to March 2016, 58 patients (35 women and 23 men, mean age 53, range 42-73) with proven anal cancer underwent clinical examination and anoscopic examination. In Table 4 we report the demographics data of enrolled patients. All patients underwent MRI and 3D EAUS study. Those subjects who underwent neoadjuvant treatment were subjected to control after therapies with both techniques (90 days on average, range 86-94 days).

**Table 4:** Patients demographics data

Description	Numbers (%)
Gender	Men 23 (39.7 %)
	Women 35 (60.3%)
Age	53 (range 42-73)
**Clinical symptoms**	
Blood in stool	58(100%)
Painful defecation	58 (100%)
Anal pain/ perianal pain	58 (100%)
Defecation and stool irregularities	58 (100%)
Pruritus	32 (55.1%)
Foreign body sensation	24 (41.4%)
Constipation	58 (100%)
Tumor on self-palpation	3 (5.2%)
Inguinal lymph nodes on self-palpation	1 (1.7%)
Systemic symptoms (Weight loss or anemia)	5 (8.6%)
History of vaginal or cervical cancer	8 (13.8%)
History of known HPV infection	12(20.7%)

### MR imaging protocol

MR Imaging was performed with a 1.5T scanner (Magnetom Symphony, Siemens Medical System, Erlangen, Germany) equipped with a phased-array body coil. Patients were placed in a supine, headfirst position. Mild rectal lumen distension was achieved with 60-90 ml of ultrasound gel or superparamagnetic contrast medium (Lumirem; Guerbet, Roissy CdG Cedex, France) introduced per rectum. Pre-contrast sagittal and axial T2 weight (W) 2D turbo spin-echo (TSE) images of the pelvis were obtained. Axial, dynamic, contrast enhanced T1W, FLASH 3D gradient-echo (GRE) images were acquired for the qualitative (q) MRI analysis (inspective analysis of TIC). We obtained one sequence before and ten sequences, without any delay, after IV injection of 2 ml/kg of a positive, gadolinium based paramagnetic contrast medium (Gd- DOTA, Dotarem, Guerbet, Roissy CdG Cedex, France). The contrast medium was injected using Spectris Solaris® EP MR (MEDRAD Inc., Indianola, PA), with a flow rate of 2 ml/s, followed by a 10-mL saline flush at the same rate. Total acquisition time for pre-contrast and ten post-contrast sequences was 6.4 minutes. Sagittal, axial and coronal post contrast T1W 2D TSE, with and without fat saturation were obtained. The details of pulse Sequence Parameters are reported in Table 5.

**Table 5:** Pulse sequence parameters

Sequence	Orientation	TR/TE/FA(ms/ms/deg.)	AT(min)	FOV(mm x mm)	AcquisitionMatrix	ST/Gap(mm/mm)	TF
T1w 2D TSE	Coronal	499/13/150	2.36	450×450	256×230	3 / 0	3
T2w 2D TSE	Sagittal	4820/98/150	4.17	260×236	256×139	3 / 0	13
T2w 2D TSE	Axial	3970/98/150	3.48	270×236	256×157	3 / 0	13
SE-DW-EPI	Axial	2700/83	6.37	136×160	160×102	4/0	/
T1w FLASH 3D	Axial	9.8/4.76/25	0.58	330×247	256×192	3 / 0	/
T1w FLASH 3D	Axial	9.8/4.76/25	0.58×10	330×247	256×192	3 / 0	/
T1w 2D TSE	Sagittal	538/13/150	2.35	250×250	256×230	3 / 0	5
T1w 2D TSE	Coronal	538/13/150	2.52	250×250	256×230	3 / 0	5
T1w 2D TSE	Axial	450/12/150	2.31	270×236	256×202	3 / 0	5

Note. TR = Repetition Time, TE = Echo Time, FOV = Field of View, FA = Flip Angle, ST = Slice Thickness, TF = Turbo Factor, AT = Acquisition Time.

### 3D-EAUS imaging protocol

The examinations were performed with a Bruel and Kjaer ProFocus system Ultra View-2202 (Mile- parken 34, 2730 Herlev, Denmark) with a model 2052 trans-ducer equipped with a double multi frequency crystal (range: 6–16 MHz), with 360° mechanical rotation at a speed of 1.9–2.8rotations/s, focus range up to 45mm, dimensions 550×270×40×17 mm, and automatic extraction and field depth up to 10 cm. All patients were examined in the lateral decubitus position without any prior bowel preparation and without any anesthesia. The transducer was covered with a condom and, after adequate lubrication, placed inside the anal canal. The transducer was firstly advanced as far as the rectal ampulla before continuing with more caudal scans; it was then automatically withdrawn to the superficial perianal plane. Images were viewed in planes perpendicular to the transducer, which was kept with the same orientation so that the anterior wall was always visualized at the 12 o'clock position, the left wall at 3 o'clock, the posterior wall at 6 o'clock, and the right wall at 9 o'clock.

Three scan planes were acquired:

(1) The deeper plane corresponded to the proximal extremity of the anal canal, where there is the typical U-shaped sling appearance of the hyperechoic puborectalis muscle with the wider end towards the pubis.

(2) The intermediate plane included the hypoechoic internal anal sphincter (IAS), the perianal body, and the transverse perianal muscle.

(3) The superficial plane corresponded to the level of the distal extremity of anal canal and included the hyperechoic layer of the submucosal portion of the external anal sphincter (EAS).

### Images analysis

3D EAUS and MR imaging analysis were done independently at different time. Two in-site observers with at least 20 and 10 years of experience of pelvic MR examination recorded all data in complete accordance. All 3D EAUS images were retrospectively analyzed by two observers, independently of each other and to avoid discrepancy, they examined the case together until agreement was reached. The radiologists evaluated the presence of lesions, using a 4-point confidence scale (score) [17]; 1, no lesion; 2, probably no lesion; 3, probably lesion; 4, definitely lesion. For each single lesion the radiologists recorded also the site, extent and distance from the anal verge, distance from the anorectal junction, the degree of infiltration of the sphincter's plane and the presence of lymph nodes at EAUS and MRI. The tumor location was classified according to the involvement of proximal anal channel (close to the rectum) and/or distal anal channel (bordering the skin). We reported the appearance of the tumor on US image, the signal intensity (SI) in T1-W images, T2-W images, in Diffusion weighted images (DWI), in the apparent diffusion coefficient (ADC) map, the value of ADC as well as the enhancement pattern during dynamic study with corresponding TIC on MRI. The appearance of the tumor on US images was defined as hypoecoic, hyperecoic or isoecoic in respect to adjacent muscle structures, so that the SI of the lesions on T1-W and T2-W images was categorized as isointense, hypointense, and hyperintense compared to surrounding muscle structures. When the lesion was hyperintense on all b values we defined this a restricted diffusion. The DW signal decay was analyzed using a linear fitting of the mono-exponential model, according to the equation ADC = ln (S0/Sb)/b, where Sb is the SI with diffusion weighting b (b>200 s/mm^2^) and S0 is the non-diffusion-weighted SI. This analysis was based on region of interest (ROI) using median value of single voxel signals for each b value. ROI for the lesion was manually drawn to include such hyperintense voxels on image at b value of 800 s/mm2. Median diffusion parameters of ROI were used as representative values for each lesion. No motion correction algorithm was used but ROIs were drawn taking care to exclude areas in which movement artifacts or blurring caused voxel misalignments. The data analysis was performed using in-house software written in Matlab (The MathWorks, Inc., Natick, USA).

Qualitative (q) Dynamic Contrasted Enhancement (DCE)-MRI involves the visual inspection and classification of TIC in accordance with the scheme proposed by Daniel et al [18]. qDCE-MRI evaluation was done by radiologists in consensus, placing multiple ROIs inside the lesions on dynamic contrast FLASH 3D GRE. Each ROIs area was of 5 pixels (0.54 × 0.54mm^2^ each pixel). We followed the scheme based on qualitative evaluation of TIC shapes proposed by [18]: type 1, no enhancement; type 2, slow sustained enhancement; type 3, rapid initial and sustained late enhancement; type 4, rapid initial and stable late enhancement; type 5, rapid initial and decreasing late enhancement.

Moreover, we reported the measure and the appearance of the perianal, perirectal, iliac and inguinal neoplastic nodes on US, SI on T2-W and T1-W images, on DWI and ADC map and the value of ADC.

In patients undergoing neoadjuvant therapy radiologists on post-treatment images re-evaluated the same parameters. For q-DCE-MRI post-treatment persistence of the same curve shape type or a change into a higher type (for example, from type 4 to type 5) was considered a negative response to treatment, while a change in a lower type (for example from type 4 to type 3 or type 2) was considered as responder to treatment [16]. For DWI analysis persistence of the same SI on b800 s/mm^2^ and ADC values on images post treatment was considered as a non responder to treatment while a SI lower or a disappearance of the lesion on b800 s/mm^2^and a percentage variation of ADC values higher than 30% was considered as responder to treatment both for lesion that for neoplastic node [19].

The gold standard was clinical, anoscopic and histological examination.

### Statistical analysis

Median values of variables before and after treatment were analysed using the non-parametric Wilcoxon and Mann–Whitney U test for paired and unpaired data, rispectively. A P value <0.05 was considered significant for all tests. All analyses were performed using Statistics Toolbox of Matlab R2007a (The Math-Works Inc., Natick, MA).
